# A comprehensive search string informed by an operational definition of complementary, alternative, and integrative medicine for systematic bibliographic database search strategies

**DOI:** 10.1186/s12906-022-03683-1

**Published:** 2022-07-27

**Authors:** Jeremy Y. Ng, Tushar Dhawan, Ekaterina Dogadova, Zhala Taghi-Zada, Alexandra Vacca, Renee-Gabrielle Fajardo, Hooriya A. Masood, Riva Patel, Samira Sunderji, L. Susan Wieland, David Moher

**Affiliations:** 1 Department of Health Research Methods, Evidence, and Impact, Faculty of Health Sciences, McMaster University, Hamilton, Ontario Canada; 2 Centre for Journalology, Clinical Epidemiology Program, Ottawa Hospital Research Institute, Ottawa, Canada; 3 Center for Integrative Medicine, University of Maryland School of Medicine, Baltimore, MD USA; 4 School of Epidemiology and Public Health, Faculty of Medicine, University of Ottawa, Ottawa, Canada

**Keywords:** Complementary and alternative medicine, Integrative medicine, Operational definition, Standard of classification, Bibliographic database, Medical research platform, Search strategy, Search string

## Abstract

**Background:**

Determining which therapies fall under the umbrella of complementary, alternative, and/or integrative medicine (CAIM) is difficult for several reasons. An operational definition is dynamic, and changes depending on both historical time period and geographical location, with many countries integrating or considering their traditional system(s) of medicine as conventional care. We have previously reported the first operational definition of CAIM informed by a systematic search. In the present study, we have developed a comprehensive search string informed by an operational definition of CAIM for systematic bibliographic database search strategies.

**Methods:**

We developed a single search string for the most common bibliographic databases, including those searchable on the OVID platform (e.g., MEDLINE, EMBASE, PsycINFO, AMED), the EBSCO platform (e.g., ERIC, CINAHL), Scopus, and Web of Science, using the finalised operational definition of CAIM’s 604 therapies. We searched the Therapeutic Research Center’s “Natural Medicines” database for all 604 therapies, and each item’s scientific name and/or synonym was included as a keyword or phrase in the search string.

**Results:**

This developed search string provides a standardised list of CAIM terms (i.e., keywords and phrases) that may be searched on bibliographic databases including those found on the OVID platform (e.g., MEDLINE, EMBASE, PsycINFO, AMED), the EBSCO platform (e.g., ERIC, CINAHL), Scopus, and Web of Science.

**Conclusion:**

Researchers can select relevant terms for their CAIM study and insert the keywords/phrases into these databases to receive all accessible data. This search technique can simply be copied and pasted into the search bar of each database to identify research by keywords, which is the most inclusive, or by words in the article title, which is more selective. Given its versatility across multiple commonly used academic platforms/databases, it is expected that this search string will be of great value to those conducting research on CAIM topics involving systematic search strategies.

**Supplementary Information:**

The online version contains supplementary material available at 10.1186/s12906-022-03683-1.

## Introduction

Complementary, alternative, and integrative medicine (CAIM) refers to non-mainstream practices that are used together with, in place of, or in coordination with conventional medicine, respectively [1]. However, the identification of a unified set of CAIM therapies presents challenges: there may be limited recognition of therapies that stand alone or exist without a complementary, alternative, or integrative relationship to conventional medicine, and there is a lack of universal consensus regarding how to accurately label a given CAIM therapy, even in the medical literature [2]. Despite these challenges, patients continue to use CAIM globally. For example, of the 170 countries that comprise the World Health Organization, 88% adopted policies and programs for CAIM [3, 4]. Patients may actively choose to use CAIM therapies in an attempt to improve physical and mental aspects of their well-being, such as achieving symptom relief and gaining a sense of control over their own care, respectively [5–7]. These patient preferences propel research efforts in the CAIM field, as new evidence allows healthcare practitioners to engage in shared decision-making with their patients [2, 8].

Since there is no standard list of CAIM therapies that is mutually agreed upon by the research community, certain research studies, including umbrella, scoping, and systematic reviews and bibliometric analyses, that aim to be comprehensive about CAIM, are methodologically limited. The failure to include a comprehensive list of CAIM therapies in a search strategy can lead to the omission of eligible research studies, which may result in a study arriving at an inaccurate conclusion. Since the 1940s, the volume of CAIM publications has followed an upward trend, with 2020 being the most productive year globally to date based on a bibliometric analysis published before the end of 2021 [9]. To continue this trend, a comprehensive operational definition, and by extension a search strategy, of CAIM is needed. So far, only one operational definition of complementary and alternative medicine (CAM) has been published to date, by Wieland et al. in 2011 [10]. However, this definition was developed for the purpose of selecting relevant systematic reviews from a single journal, did not consider the “integrative” component of CAIM, considered only two sources (the Medical Subject Headings (MeSH) definition of complementary therapies and the Complementary Medicine subset search strategy in PubMed) as potential sources of an operational definition, and was not designed to result in a search strategy [10]. Nevertheless, Wieland et al.’s sentiment of supporting the harmonization of CAIM research with a more standardized and precise classification of CAIM therapies that would enable better collaboration among researchers, clinicians, and others who seek a comprehensive picture of CAIM is shared by the present study. Thus, the objective of this study is to construct a comprehensive search string informed by an operational definition of CAIM – which we have described in a separate publication – for systematic bibliographic database search strategies.

## Methods

### Previous study: Operational definition of CAIM

We have previously reported the first operational definition of CAIM informed by a systematic search, which serves as the basis for the present study [11]. Briefly, the operational definition of CAIM was derived from four types of quality-assessed media types: 1) peer-reviewed articles from the MEDLINE, EMBASE, AMED, PsycINFO, CINAHL, Scopus and Web of Science databases; 2) the “Aims and Scopes” of peer-reviewed CAIM journals; 3) CAIM entries in highly accessed online encyclopaedias; and 4) websites resulting from Health On the Net Code of Conduct (HONcode) searches. Across these four media types, eligible items were reviewed for CAIM or CAIM-related terms and data extracted for the operational definition. Next, we grouped similar/identical CAIM therapies (e.g., “St. John’s wort” and “*Hypericum perforatum*”) together to appear on a single line after finalising the list of CAIM therapies contained in the operational definition, then alphabetized all lines. We also searched for all common and scientific names for each CAIM therapy in monographs produced by the Natural Medicines Research Collaboration [12]; in cases where no professional monograph was available, we consulted the peer-reviewed literature. The Methods section of our previous study describes our eligibility criteria, searching and screening, data extraction and analysis, and the creation of an operational definition of CAIM. The final operational definition of CAIM is available here: https://bmccomplementmedtherapies.biomedcentral.com/articles/10.1186/s12906-022-03556-7/tables/2. A protocol was registered with the Prospective Register of Systematic Reviews (PROSPERO), registration number CRD42020206301.

### Development of the bibliographic database search string

We developed a single search string for the most commonly used bibliographic databases including those searchable on the OVID platform (e.g., MEDLINE, EMBASE, PsycINFO, AMED), the EBSCO platform (e.g., ERIC, CINAHL), as well as Scopus and Web of Science, using the 604 therapies in the finalized operational definition of CAIM. To accomplish this, we added every relevant scientific name and/or synonym found in the monograph as a term (i.e., keyword, phrase) to the search string. By search string, we refer to a combination of keywords/phrases and boolean operators that can be entered into the search box of a bibliographic database, which comprises a single line of a search strategy. We opted to exclude controlled vocabularies from our search string for two main reasons: 1) this would require customized search strings for each database, and 2) this would be impractical, given that many databases regularly update their indexing terms (for example, MEDLINE updates their MeSH terms annually: https://www.nlm.nih.gov/mesh/whatsnew.html). We also reviewed common features that are recognized across all the aforementioned platforms/databases, opting to use asterisks (*) as opposed to other wildcard symbols. The “OR” Boolean operator was used to separate each individual keyword/phrase included in the search string.

To improve specificity, we carried out a series of tests with respect to term-specific searches, followed by observing and evaluating search results yielded, and subsequently modifying the search string. As a result of this, we identified and removed terms as follows: 1) acronyms (e.g. “TENS” as an acronym for “transcutaneous electrical nerve stimulation”); 2) element symbols (e.g., “Fe” as the symbol for “iron”); and 3) amino acid 3 letter codes (e.g., “Arg” as the code for “arginine”), as it is generally not common practice to include just the acronyms, element symbols, or amino acid 3 letter codes alone in titles and abstracts. Lastly, we also identified and removed 4) other terms that had the potential to generate a disproportionate volume of non-CAIM-specific search results (e.g., “May” as a synonym for “Hawthorn” (*Crataegus* spp.)); these terms were identified through standalone searches conducted on MEDLINE whereby the first line of results was visually scanned for a large proportion of non-CAIM-specific search results. Before terms were excluded, we searched them on multiple databases and reviewed the first page of results to verify that non-CAIM-specific search results were being retrieved.

### Review by information specialist

We acknowledge that the involvement of an information specialist is key for comprehensive identification of relevant evidence and is a hallmark of research quality [13], and therefore, we recruited an academic librarian with considerable experience in developing and reviewing systematic search strategies. The academic librarian provided critical feedback on our search string, ran our searches on the bibliographic databases to verify its usability, and reviewed the terminology used to report our findings in this article.

## Results

### Bibliographic database search string

This formulated bibliographic database search string provides a standardized list of CAIM terms that is searchable on frequently used databases including those found on the OVID platform (e.g., MEDLINE, EMBASE, PsycINFO, AMED), the EBSCO platform (e.g., ERIC, CINAHL), as well as Scopus and Web of Science. Searchers may identify the terms that are relevant to their CAIM focus and enter the terms on these databases to retrieve all available data. This search string can simply be pasted into each database’s search field to identify studies by keywords, which is most inclusive, or by words in the article title, which is more selective. The search string is available for download as Supplementary File 1. When searching the search string in a given bibliographic database or platform, it is recommended to use the respective “advanced search” feature; in most cases, it can simply be copied and pasted directly into the advanced search bar and exist as a single line of the entire search strategy. Additionally, and for the purpose of increased transparency, a complete list of all excluded terms and their corresponding reason for removal is available for download as Supplementary File 2.

## Discussion

In the present study, one exhaustive search string using terms (i.e., keywords, phrases), was developed based on the operational definition of CAIM we reported in our previous study. Prior to this, Wieland et al. (2011) developed an operational definition of CAM consisting of 70 therapies [10] and the Cochrane Complementary Medicine website later amplified this by listing 259 CAIM therapy terms [14]. Our previously reported study represents the largest operational definition of CAIM created to date, with 604 CAIM therapy terms and their respective synonyms; in addition, it is the only systematically and transparently developed operational definition of its kind [11]. The present study is the first to develop and report a comprehensive search string informed by an operational definition of CAIM for systematic bibliographic database search strategies.

Umbrella, scoping, and systematic reviews, as well as bibliometric analyses, are areas in which a comprehensive CAIM search string would be of great utility for inclusion in a systematic search strategy. These are research methodologies that gather literature published in a certain academic discipline, either to fill a knowledge gap for systematic and scoping reviews, or to identify characteristics of the literature, such as the impact factor, for bibliometric analyses [15–25]. Prior to the publication of this search string, different approaches have been taken by different researchers to capture CAIM-related literature for systematic and scoping reviews, which introduced selection biases into their search results [26]. Likewise, bibliometric analyses generally assessed CAIM research from CAIM journals [27, 28], based on research methodologies [29, 30], specific CAIM interventions [31–36], or one or more CAIM-related indexed headings (e.g., MeSH) [37, 38], omitting additional studies that might have been captured with a comprehensive list of CAIM therapies. Additionally, it is worth mentioning that the PubMed Dietary Supplement Subset has been discontinued as of April 2020 and is no longer available [39], and the search strategy used to create the PubMed Complementary Medicine Filter has not been updated since 2019 [40]. Given the lack of regularly updated standardized search strategies based on keywords/phrases and indexed headings, an approach based on comprehensive identification of CAIM therapy terms may serve as an inclusive tool for identifying relevant studies. Fortunately, standardized academic search strategies allow researchers to obtain more consistent search results and consequently find a plethora of relevant peer-reviewed literature and accurately gauge the current status, as well as the future directions, of CAIM research in their respective fields [41–45]. It can be anticipated that should researchers conducting future reviews and analyses use the search string presented in this study in their search strategies, they will identify a more comprehensive collection of eligible general CAIM studies or literature characteristics.

### Running the search strategy: considerations

To run this search string provided in Supplementary File 1 in a bibliographic database, it can simply be copied/pasted into a given database’s search field, accompanied by the appropriate floating subheading where applicable (e.g., “.ti” for a title search in MEDLINE). Following testing the running of this search string on the aforementioned bibliographic databases, users should be aware, however, that this search string contains keywords/phrases originating from over 600 CAIM therapies and is, therefore, very long. A search string with this many terms may take a few minutes to load. Furthermore, should the search time out or fail to run, we recommend that users break up the string across multiple search lines, then combine the results by applying the “OR” function. We also acknowledge that not all search terms included in this search string may be relevant for every search depending on the aims and objectives of a given study. Users may consider removing terms irrelevant to their study’s topic. Regardless of whether users elect to remove terms or not, once the search has been run, with all terms included, users should not be alarmed by the fact that this may result in > 1 million results. Given that any study objective will include an additional intersecting topic (e.g., cancer), users may succeed in eliminating irrelevant search results simply by introducing other search lines relating to their intersecting topic and applying the “AND” function (e.g., CAIM search strategy “AND”-ed with search lines containing cancer-related terms [assuming the authors are interested in recovering articles at the intersection of CAIM and cancer]). Further to this, users may consider applying search filters made available by the InterTASC Information Specialists’ Sub-Group Search Filter Resource; it is a “collaborative venture to identify, assess, and test search filters designed to retrieve research by study design or focus” [46]. Published literature has found that much of the usefulness of keywords stems from the simultaneous presence of controlled vocabularies [44, 47]. Thus, one solution that we propose for users concerned that the present search string may not capture results yielded by the use of indexed headings is to consider applying both as separate search lines in their search strategy.

### Challenges and limitations

Despite its utility, there are challenges and limitations using this search string to seek out general CAIM literature. There are various frameworks regarding what constitutes CAIM across different countries and cultures; thus, the search string may result in under- or over-inclusiveness of certain therapies as there is still no consensus, even among experts, regarding what constitutes a CAIM therapy [10, 26]. For example, CAIM therapies that originate from Buddhist practices may be considered part of conventional care in Eastern countries such as Thailand and Sri Lanka, while European and North Americans countries may consider these practices as CAIM [48, 49]. Similarly, there is also an English language bias, as the search string was derived from the operational definition which was constructed based on English literature only. The majority of CAIM studies are published in the English or Chinese languages and interestingly, English journals have been found to deviate towards reporting the negative results of CAIM use, while Chinese journals report positive results [30, 50, 51]. As a result, the language of publication may influence which therapies are discussed by a study, and consequently which were included in our list [52, 53].

One limitation inherent to our search string in general also exists but is augmented by the dynamic nature of the CAIM field. There is a trade-off between recall and precision in terms of results retrieval. If a search term is too ambiguous or broad, there will likely be “noise” that researchers will have to sift through [45]. Alternatively, if a search term is too specific or narrow, eligible articles may be missed. Lastly, we acknowledge that while keywords/phrases are searched in all database fields, and thus, are flexible and broad approaches to searching, they may suffer both from the problem of synonyms and variants, resulting in missing relevant records, and from the presence of article terms that are not the main focus of the research, resulting in over-retrieval of irrelevant results [54]. Searches including indexed headings may provide more targeted results and may also provide access to related material through index hierarchies. On the other hand, indexing may or may not capture all relevant concepts of interest; while MEDLINE, for example, currently uses automated indexing based on title and abstract to rapidly index the majority of database records, full indexing by humans can take several months [55].

We argue that by having made the decision to carefully review and remove search terms resulting from our operational definition of CAIM that fell into one of the four aforementioned categories, this ultimately provided a better balance between sensitivity and specificity. While we have prioritized optimizing both recall and precision, the differences in CAIM therapy description by country, culture, language, system of traditional medicine, and school of thought, looms, nevertheless. Lastly, we acknowledge that while we did involve an academic librarian in reviewing this study, the involvement of additional information specialists may have provided us with more points of improvement; we plan to involve additional information specialists in future research that expands on our present work.

### Future directions

While we anticipate that the search string provided in this study will provide considerable utility, we also propose several future directions based on the challenges and limitations already outlined. It would be of value to understand whether the present search string informed by an operational definition of CAIM better captures the CAIM literature than other available search alternatives (e.g., PubMed Complementary Medicine Filter [40]). One of the obvious advantages of the search string we present, is that it is applicable to bibliographic databases beyond PubMed, which is particularly important for CAIM. A search for other CAIM-related search strings and/or strategies online yield some results [56–58], however, they have been published even prior to the first operational definition presented by Wieland et al. in 2011 [10]. Other available search strings include CAIM-related indexed headings themselves; such examples include “exp Complementary Therapies/” (~ 180,000 results as of April 2022) and “exp Integrative Medicine/” (~ 2000 results) in MEDLINE [59], and “exp alternative medicine/” (~ 70,000 results) and “exp integrative medicine/” (~ 6000 results) in EMBASE [60]. From the number of results generated based on these searches, however, the number of results yielded by indexed heading searches pale in comparison to those yielded by the search string informed by our operational definition of CAIM (> 1 million search results). Another future direction includes identifying what modifications, if any, can improve the sensitivity and/or specificity of the search string. One idea which may serve to achieve this goal, at least in part, for example, could involve the use of a selection of keywords/phrases contained within the search strategy to create a series of CAIM category-specific search strings (e.g., nutritional, [inclusive of keywords/phrases relating to special diets, dietary supplements, herbs, and probiotics]; psychological [inclusive of keywords/phrases relating to mindfulness]; and physical [inclusive of keywords/phrases relating to massage, and spinal manipulation]) [1]. Further to this, future research, perhaps conducted in specific areas of CAIM, should consider the comparative retrieval of keyword/phrase-, indexed heading-, and combined keyword/phrase and indexed heading-based searches to further clarify the benefits and limitations of the approaches we have proposed. Finally, we encourage librarians and other information specialists to evaluate our search string in order to iteratively improve it.

## Conclusion

In this study, we have created the first exhaustive search string to support systematic searching in CAIM research, based on our previous work which developed the first operational definition of CAIM informed by a systematic search of quality-assessed media sources. Determining which therapies fall under the umbrella of CAIM is difficult for a plethora of reasons. Many countries are integrating or viewing their traditional system(s) of medicine as conventional care, and an operational definition is dynamic and fluctuates depending on both historical time period and geographical region. The search string presented in this study provides a standardised list of CAIM terms that can be executed on commonly used bibliographic databases. We anticipate that this published search string will be of considerable value to any researcher conducting a study on the topic of CAIM involving systematic searches, due to its versatility across various commonly used academic platforms/databases. Lastly, we welcome testing of our search string by librarians, among other information specialists, to improve it iteratively.

## Supplementary Information

**Additional file 1: Supplementary File 1.** Comprehensive Search String Derived from an Operational Definition of Complementary, Alternative, and Integrative Medicine

**Additional file 2: Supplementary File 2.** List of CAIM-related Terms Removed from Comprehensive Search String

## Data Availability

All relevant data are included in this manuscript.

## Abbreviations

CAIM: Complementary, Alternative, and Integrative Medicine
CAM: Complementary and Alternative Medicine
HONcode: Health On the Net Code of Conduct
MeSH: Medical Subject Headings
PROSPERO: Prospective Register of Systematic Reviews
