# Supplementary material for: A comprehensive search string informed by an operational definition of complementary, alternative, and integrative medicine for systematic bibliographic database search strategies
Source: BMC Complement Med Ther. 2022 Jul 27;22:200. doi: 10.1186/s12906-022-03683-1 (PMC9327196; doi:10.1186/s12906-022-03683-1)
Supplement: Supplementary file 2 — Additional file 2: Supplementary File 2. List of CAIM-related Terms Removed from Comprehensive Search String [file 12906_2022_3683_MOESM2_ESM.docx]

**Supplementary File 2: List of CAIM-related Terms Removed from Comprehensive Search String**

| **Term** | **Reason for Removal** |
| --- | --- |
| AC-1202 | Acronym |
| ACPI | Acronym |
| ADH | Acronym |
| AGE | Acronym |
| AGPI | Acronym |
| AHAE | Acronym |
| AHE | Acronym |
| Ail | Has the potential to generate a disproportionate volume of non-CAIM-specific search results |
| ALC | Acronym |
| ALCAR | Acronym |
| Andro | Has the potential to generate a disproportionate volume of non-CAIM-specific search results |
| Anti-TAF | Acronym |
| Arg | Amino acid 3 letter code |
| Awa | Has the potential to generate a disproportionate volume of non-CAIM-specific search results |
| Bal | Has the potential to generate a disproportionate volume of non-CAIM-specific search results |
| Bay | Has the potential to generate a disproportionate volume of non-CAIM-specific search results |
| BC-PS | Acronym |
| BFR | Acronym |
| BFRs | Acronym |
| Bol | Has the potential to generate a disproportionate volume of non-CAIM-specific search results |
| Bore | Has the potential to generate a disproportionate volume of non-CAIM-specific search results |
| Boro | Has the potential to generate a disproportionate volume of non-CAIM-specific search results |
| BT | Acronym |
| BTC | Acronym |
| Bud | Has the potential to generate a disproportionate volume of non-CAIM-specific search results |
| CBD | Acronym |
| CDS | Acronym |
| CFA | Acronym |
| CFAs | Acronym |
| Chih | Has the potential to generate a disproportionate volume of non-CAIM-specific search results |
| CLA | Acronym |
| CM | Acronym |
| CMO | Acronym |
| CoQ10 | Acronym |
| CPS | Acronym |
| CPTV | Acronym |
| Cr III | Acronym |
| Cr3+ | Acronym |
| CS | Acronym |
| Cs-4 | Acronym |
| CSA | Acronym |
| CSC | Acronym |
| CST | Acronym |
| CTTFT | Acronym |
| Cu | Element Symbol |
| DHT | Acronym |
| DLPA | Acronym |
| DMPS | Acronym |
| DMSO | Acronym |
| Dope | Has the potential to generate a disproportionate volume of non-CAIM-specific search results |
| DSC | Acronym |
| E-EPA | Acronym |
| EDT | Acronym |
| EFT | Acronym |
| EGCG | Acronym |
| EPO | Acronym |
| EQT | Acronym |
| Fe | Element Symbol |
| Fer | Has the potential to generate a disproportionate volume of non-CAIM-specific search results |
| FL-113 | Has the potential to generate a disproportionate volume of non-CAIM-specific search results |
| G6S | Has the potential to generate a disproportionate volume of non-CAIM-specific search results |
| GAG | Acronym |
| GCP | Acronym |
| GDV | Acronym |
| Gea | Has the potential to generate a disproportionate volume of non-CAIM-specific search results |
| GI | Has the potential to generate a disproportionate volume of non-CAIM-specific search results |
| Gln | Amino acid 3 letter code |
| Gobo | Has the potential to generate a disproportionate volume of non-CAIM-specific search results |
| GS | Has the potential to generate a disproportionate volume of non-CAIM-specific search results |
| GTF | Acronym |
| GTF-Cr | Acronym |
| GTP | Acronym |
| GTPF | Acronym |
| Hash | Has the potential to generate a disproportionate volume of non-CAIM-specific search results |
| HAW | Has the potential to generate a disproportionate volume of non-CAIM-specific search results |
| HBOT | Acronym |
| HEP-30 | Acronym |
| HT | Acronym |
| HupA | Has the potential to generate a disproportionate volume of non-CAIM-specific search results |
| I | Element Symbol |
| I2 | Has the potential to generate a disproportionate volume of non-CAIM-specific search results |
| IAT | Acronym |
| IER | Acronym |
| JM | Acronym |
| Joint | Has the potential to generate a disproportionate volume of non-CAIM-specific search results |
| K | Element Symbol |
| K1 | Has the potential to generate a disproportionate volume of non-CAIM-specific search results |
| K2 | Has the potential to generate a disproportionate volume of non-CAIM-specific search results |
| K3 | Has the potential to generate a disproportionate volume of non-CAIM-specific search results |
| K4 | Has the potential to generate a disproportionate volume of non-CAIM-specific search results |
| K5 | Has the potential to generate a disproportionate volume of non-CAIM-specific search results |
| KAO | Has the potential to generate a disproportionate volume of non-CAIM-specific search results |
| KI | Has the potential to generate a disproportionate volume of non-CAIM-specific search results |
| Kif | Has the potential to generate a disproportionate volume of non-CAIM-specific search results |
| LA | Acronym |
| LCD | Acronym |
| LCD | Acronym |
| LCHPD | Acronym |
| LCHPD | Acronym |
| LDT | Acronym |
| LECI-PS | Acronym |
| LEDs | Acronym |
| LI 132 | Acronym |
| LI132 | Acronym |
| LT | Acronym |
| Lys | Amino acid 3 letter code |
| May | Has the potential to generate a disproportionate volume of non-CAIM-specific search results |
| MBRP | Acronym |
| MBSR | Acronym |
| MCHA | Acronym |
| MCHC | Acronym |
| MCTs | Acronym |
| MDT | Acronym |
| MeDi | Has the potential to generate a disproportionate volume of non-CAIM-specific search results |
| MEL | Acronym |
| MFT | Acronym |
| MIRE | Acronym |
| MK-1 | Acronym |
| MK-10 | Acronym |
| MK-11 | Acronym |
| MK-12 | Acronym |
| MK-13 | Acronym |
| MK-2 | Acronym |
| MK-4 | Acronym |
| MK-5 | Acronym |
| MK-6 | Acronym |
| MK-7 | Acronym |
| MK-8 | Acronym |
| MK-9 | Acronym |
| MLT | Acronym |
| MMF | Acronym |
| Mn | Element Symbol |
| Mo | Element Symbol |
| MSI-1256F | Acronym |
| MT | Acronym |
| N-A-G | Acronym |
| NAG | Acronym |
| ND | Acronym |
| NMT | Acronym |
| NOP | Acronym |
| NSC-763 | Acronym |
| OFPA | Acronym |
| OLT | Acronym |
| OPC | Acronym |
| OPCs | Acronym |
| p-GlcNAc | Acronym |
| P57 | Acronym, Has the potential to generate a disproportionate volume of non-CAIM-specific search results |
| PCO | Acronym |
| PCOs | Acronym |
| PE | Acronym |
| PEMF | Acronym |
| PEMT | Acronym |
| Pepe | Has the potential to generate a disproportionate volume of non-CAIM-specific search results |
| pGlcNAc | Acronym |
| PLE | Acronym |
| Pot | Has the potential to generate a disproportionate volume of non-CAIM-specific search results |
| PRT | Has the potential to generate a disproportionate volume of non-CAIM-specific search results |
| PS | Acronym |
| PSK | Has the potential to generate a disproportionate volume of non-CAIM-specific search results |
| PSP | Acronym |
| PTV | Has the potential to generate a disproportionate volume of non-CAIM-specific search results |
| PU | Has the potential to generate a disproportionate volume of non-CAIM-specific search results |
| Q | Has the potential to generate a disproportionate volume of non-CAIM-specific search results |
| QG | Acronym |
| QI | Acronym |
| Religion | Has the potential to generate a disproportionate volume of non-CAIM-specific search results |
| RSV | Acronym |
| RSVL | Acronym |
| RT | Has the potential to generate a disproportionate volume of non-CAIM-specific search results |
| RY | Has the potential to generate a disproportionate volume of non-CAIM-specific search results |
| SAM | Acronym |
| SAMe | Acronym |
| Sammy | Has the potential to generate a disproportionate volume of non-CAIM-specific search results |
| Sang | Has the potential to generate a disproportionate volume of non-CAIM-specific search results |
| SCBI | Has the potential to generate a disproportionate volume of non-CAIM-specific search results |
| Se | Element Symbol |
| SG | Acronym |
| SJW | Acronym |
| SKY | Has the potential to generate a disproportionate volume of non-CAIM-specific search results |
| SMT | Acronym |
| SNI | Acronym |
| SO | Acronym |
| SQ-9453 | Has the potential to generate a disproportionate volume of non-CAIM-specific search results |
| SSKI | Acronym |
| ST-200 | Has the potential to generate a disproportionate volume of non-CAIM-specific search results |
| TAES | Acronym |
| TAM | Acronym |
| TAM | Acronym |
| TC-80 | Acronym |
| TCM | Acronym |
| TCMs | Acronym |
| TEAS | Acronym |
| TECA | Has the potential to generate a disproportionate volume of non-CAIM-specific search results |
| TENS | Acronym |
| THC | Acronym |
| The | Has the potential to generate a disproportionate volume of non-CAIM-specific search results |
| TIM | Acronym |
| TJM | Acronym |
| TPI | Acronym |
| TTFCA | Has the potential to generate a disproportionate volume of non-CAIM-specific search results |
| Tyr | Amino acid 3 letter code |
| UV | Acronym |
| UVA | Acronym |
| V | Element symbol |
| VT | Acronym |
| XZK | Acronym |
| ZB | Acronym |
